# Synthetic Notch CD123 and CLL1 chimeric antigen receptor T-cells (CAR-T) target acute myeloid leukemia and reduce off-tumor effects associated with CLL1 CAR-T cells

**DOI:** 10.1186/s43556-026-00598-y

**Published:** 2026-10-16

**Authors:** Rui Zhang, Na Chen, Mengnan Li, Qingxi Liu, Mingfeng Zhao

**Affiliations:** 1 Department of Hematology, Tianjin First Central Hospital, Tianjin, 300380 China; 2 First Central Hospital of Tianjin Medical University, Tianjin Medical University, Tianjin, China; 3 School of Medicine, Nankai University, Tianjin, China; 4 Qilu Institute of Technology, Shandong, China

**Keywords:** Acute myeloid leukemia, SynNotch, CD123, C-type lectin-like 1 (CLL1), Chimeric antigen receptor T (CAR-T)

## Abstract

Chimeric antigen receptor T-cell (CAR-T) therapy targeting the C-type lectin-like molecule-1 (CLL1) antigen has emerged as a promising therapeutic approach for acute myeloid leukemia (AML). However, its clinical translation is hindered by on-target, off-tumor toxicity, particularly severe neutropenia caused by CLL1 expression on normal neutrophils. To address this limitation, we constructed a synthetic Notch (SynNotch) dual-antigen AND-gated CAR-T system targeting CD123 and CLL1 to improve tumor specificity and reduce off-tumor toxicity. CD123 and CLL1 expression was first assessed in AML cell lines and primary AML blasts by flow cytometry. The in vitro cytotoxicity, serial killing activity, cytokine secretion, exhaustion-associated protein markers, and transcriptomic profiles of engineered CAR-T cells were then evaluated, and therapeutic efficacy and safety were further examined in AML xenograft models. SynNotch CD123 and CLL1 CAR-T cells showed stronger cytotoxic activity against AML blasts while exhibiting reduced activity toward normal neutrophils. In addition, these cells displayed lower expression of exhaustion-associated genes and markers, together with increased expression of genes related to naive/memory differentiation. In vivo, this CAR-T variant showed improved expansion, longer persistence, and stronger anti-leukemic activity, together with less severe neutropenia. Increased cytokine production in vitro indicated stronger T-cell activation but also suggested the need for careful evaluation of potential cytokine-related toxicity. These findings indicate that SynNotch dual-antigen-gated CAR-T cells may provide a more specific and potentially safer therapeutic strategy for relapsed/refractory AML.

**Supplementary Information:**

The online version contains supplementary material available at https://doi.org/10.1186/s43556-026-00598-y.

## Introduction

Acute myeloid leukemia (AML) represents an aggressive hematological cancer associated with frequent relapse and substantial mortality. Current standard treatment modalities include conventional chemotherapy, molecularly targeted therapies, and hematopoietic stem cell transplantation (HSCT) [1]. Although HSCT remains an important therapeutic approach, its efficacy is often limited by transplant-related complications and donor compatibility [2]. Chimeric antigen receptor T-cell (CAR-T) therapy has emerged as a highly promising approach for B-cell malignancies but has encountered major challenges in AML. Several preclinical studies have identified potential AML-associated antigens, such as CD33, C-type lectin-like 1 (CLL1), and CD70; however, their expression on normal hematopoietic cells limits their therapeutic specificity [3]. Targeting these antigens individually with CAR-T cells carries a substantial risk of on-target, off-tumor toxicity, resulting in unintended injury to healthy tissues [4]. Therefore, strategies that improve CAR-T-cell cytotoxicity against AML while sparing normal cells are urgently needed.

The synthetic Notch (SynNotch) receptor system represents a newer generation of CAR-T engineering that enables precise, logic-gated regulation of T-cell activation [5]. These cells are engineered with synthetic receptors that can be activated in response to specific antigenic signals. After recognition of a priming antigen on tumor cells, SynNotch receptors induce downstream signaling and drive the expression of a second therapeutic receptor or effector molecule [6]. This design allows CAR expression and T-cell activation to be restricted to cells with a defined antigenic profile. Recent advances in SynNotch CAR-T technology have shown promising effects in several tumor models. For example, SynNotch-based systems have demonstrated long-term anti-tumor effects in glioblastoma models [7] and improved specific targeting in metastatic neuroblastoma compared with conventional CAR-T cells [8, 9]. SynNotch receptors have also been reported to enhance anti-tumor efficacy by promoting localized cytokine secretion in a target-dependent manner [10]. In another study, SynNotch receptors selectively induced ROR1 CAR expression within the tumor microenvironment, leading to significant tumor regression without evident toxicity to normal tissues [11].

Unlike conventional CAR-T cells that are activated after recognition of a single antigen, SynNotch CAR-T cells can be engineered to require sequential or combined recognition of two distinct antigens, thereby functioning as an “AND” logic gate. Recognition of the priming antigen activates the SynNotch receptor, inducing the expression of a second CAR directed against a complementary target antigen. Consequently, full T-cell activation is restricted to cells that co-express both antigens, thereby enhancing tumor specificity and reducing on-target, off-tumor toxicity. This dual-antigen gating strategy has shown potential for improving tumor specificity and reducing off-tumor toxicity in solid tumor models, including glioblastoma and neuroblastoma. However, its application in AML remains relatively underexplored. CLL1 is a validated AML-associated antigen, and CLL1 CAR-T therapy has shown anti-leukemic activity in clinical studies. Nevertheless, its clinical use is limited by on-target, off-tumor toxicity, particularly severe and persistent neutropenia caused by CLL1 expression on normal neutrophils. In our cohort of 59 AML patients receiving CLL1 CAR-T therapy, nearly all patients developed grade 3/4 granulocytopenia, and infectious complications were frequently observed. CD123 is another antigen highly expressed on primary AML cells, including leukemic stem cells (LSCs), and co-expression with CLL1 on AML cells is observed in approximately 80% of patients. Importantly, CD123 is not expressed on mature neutrophils, making it a suitable priming antigen for restricting CLL1 CAR activation to AML cells while sparing normal granulocytes.

Collectively, these clinical observations from CLL1 CAR-T monotherapy, the differential antigen expression profiles of CD123 and CLL1 in primary AML specimens, and the tissue-selective regulatory potential of SynNotch logic-gated CAR platforms in solid malignancies jointly support the study hypothesis. We designed a CD123-primed SynNotch AND-gate circuit to conditionally trigger CLL1 CAR expression only in CD123 and CLL1 double-positive leukemic cells, which was intended to reduce the clinically important myelotoxicity associated with single-antigen CLL1 CAR-T products. Through integrated in vitro functional assays, transcriptome profiling, and in vivo xenograft validation, four anticipated features of this dual-target CAR-T construct were evaluated: enhanced direct and serial cytotoxicity against both AML cell lines and patient-derived primary blasts; reduced killing of mature normal neutrophils, with the aim of mitigating severe neutropenia; reduced T-cell exhaustion-associated signatures and increased naïve/memory-associated differentiation signatures that may support sustained anti-tumor function; as well as prolonged in vivo expansion and persistence, durable leukemia clearance, and an improved safety profile in immunodeficient mouse models. This layered antigen-recognition strategy may help address the trade-off between anti-leukemic potency and hematopoietic toxicity that restricts current single-target CAR-T therapies for AML. Distinct from simple bicistronic co-expression dual CAR systems, the hierarchical SynNotch activation mode provides spatial and temporal control of CAR expression that depends on tumor-specific dual-antigen co-occurrence, offering a programmable immunotherapy framework for relapsed/refractory AML in which myelosuppression contributes to a high risk of infection. The systematic preclinical evidence presented in this work also provides a preclinical basis for further translational evaluation of SynNotch dual-antigen CAR-T modalities targeting myeloid malignancies.

## Results

### Patient responses and complications observed in CLL1 CAR-T therapy

**Fig. 1:**
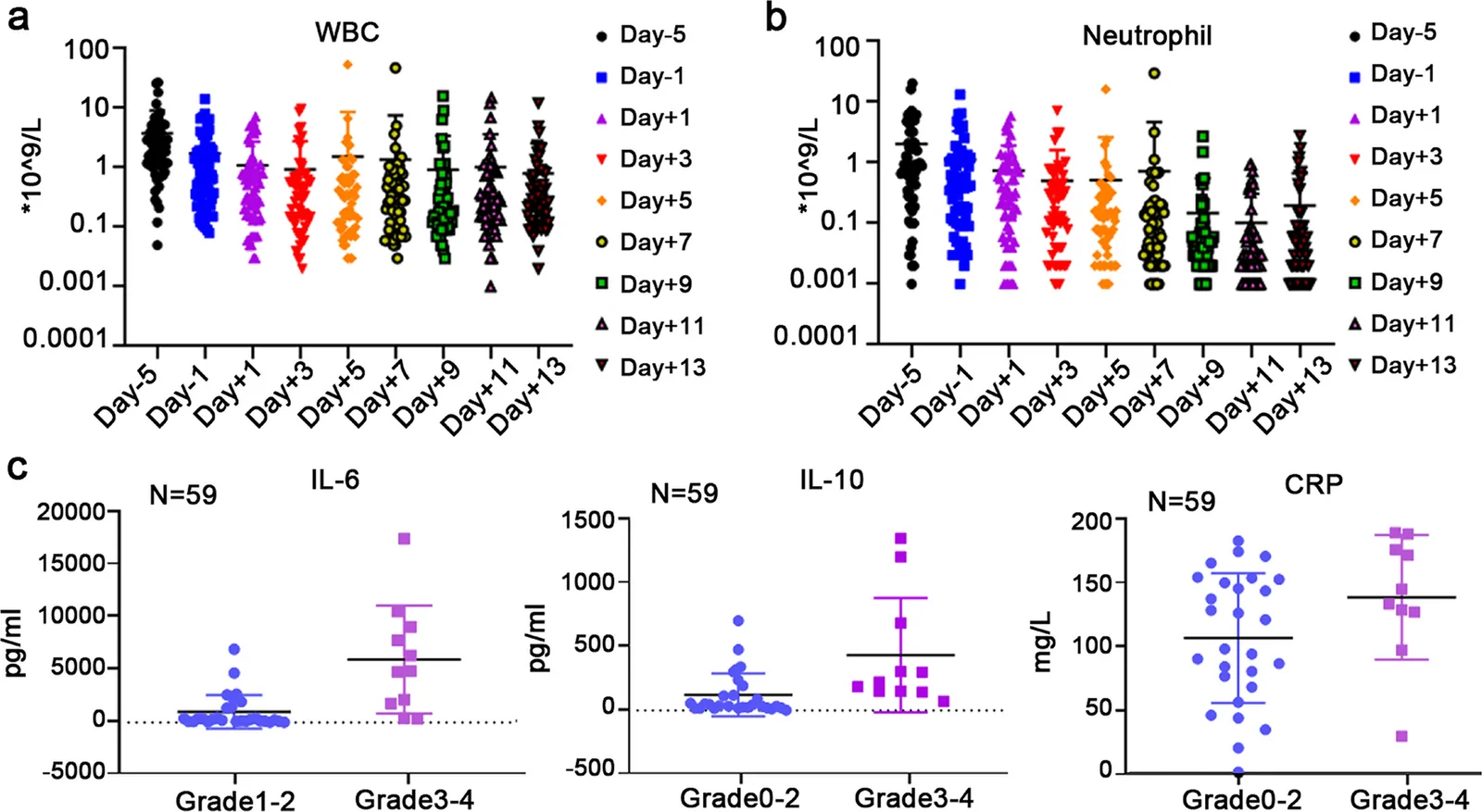
Clinical response and Cytokine Release Syndrome (CRS) in patients with relapsed/refractory acute myeloid leukemia (*n* = 59) following CLL1 CAR-T therapy. **a**, **b** Kinetics of white blood cell (WBC) and neutrophil counts post CLL1 CAR-T infusion. Data are presented as mean ± standard deviation (SD). The dashed vertical line at day 0 indicates the time of CAR-T cell infusion. **c** Peak serum concentrations of IL-10, IL-6, and CRP were compared between patients experiencing grade 1–2 and grade 3–4 CRS. Cytokine levels were measured at the peak of CRS symptoms. Data are shown as box and whisker plots: The box spans the interquartile range (IQR, 25th to 75th percentile), with the horizontal line inside the box denoting the median; The whiskers extend to the minimum and maximum values within 1.5 × IQR. Differences between groups were evaluated using the Mann–Whitney U test

Clinical data were collected from 59 patients who received CLL1 CAR-T-cell therapy. Responses were assessed 14–16 days after infusion (Table 1). Complete remission (CR) was observed in 41 of 59 individuals (69.49%); among them, 16 achieved minimal residual disease (MRD)-positive CR, while 25 achieved MRD-negative CR. The remaining 18 patients (30.51%) did not respond to therapy. Safety assessment showed that 16.95% of patients developed immune effector cell-associated neurotoxicity syndrome (ICANS), whereas cytokine release syndrome (CRS) occurred in 94.92% of patients. Severe (grade 3/4) cases accounted for 35.59% of CRS and 8.47% of ICANS. Hematologic toxicity was common: leukopenia, granulocytopenia, anemia, and thrombocytopenia were observed in most patients. White blood cell (Fig. 1a) and neutrophil (Fig. 1b) counts monitored during the first 14 days after infusion revealed persistent and profound granulocytopenia in most patients, which substantially increased the risk of infection. During the treatment course, 43 patients developed bacterial infections, 12 had viral infections, and 17 experienced fungal infections. Further analysis revealed that patients who developed severe CRS (grade 3/4) exhibited significantly higher levels of cytokine release compared with those with mild CRS (grade 1/2), characterized by markedly elevated levels of C-reactive protein (CRP), interleukin-6 (IL-6), and interleukin-10 (IL-10) (Fig. 1c).

**Table 1:** Remission rates and major adverse effects observed following CLL1 CAR-T therapy

	Total (*N* = 59)	Grade 1/2	Grade 3/4
Remission rate			
CR, n (%)	41 (69.49%)		
MRD positive CR, n (%)	16 (27.12%)		
MRD negative CR, n (%)	25 (42.37%)		
No remission (NR), n (%)	18 (30.51%)		
Side effects			
CRS, n (%)	56 (94.92%)	35 (59.32%)	21 (35.59%)
ICANS, n (%)	10 (16.95%)	5 (8.47%)	5 (8.47%)
Leukopenia, n (%)	59 (100.00%)	2 (3.39%)	57 (96.61%)
Granulocytopenia, n (%)	59 (100.00%)	1 (1.69%)	58 (98.30%)
Anemia, n (%)	54 (91.53%)	22 (37.29%)	32 (54.24%)
Thrombocytopenia, n (%)	56 (94.91%)	19 (32.20%)	37 (62.71%)
Infection, n (%)	51 (86.44%)		
Bacteria, n (%)	43 (72.88%)		
Virus, n (%)	12 (20.34%)		
Fungus, n (%)	17 (28.81%)		

*CAR-T* chimeric antigen receptor T cell, *CR* complete remission, *MRD* minimal residual disease, *CRS* cytokine release syndrome, *ICANS* immune effector cell-associated neurotoxicity syndrome

### Screening SynNotch “AND” logic-gating targets to reduce off-tumor effects of CLL1 CAR-T

**Fig. 2:**
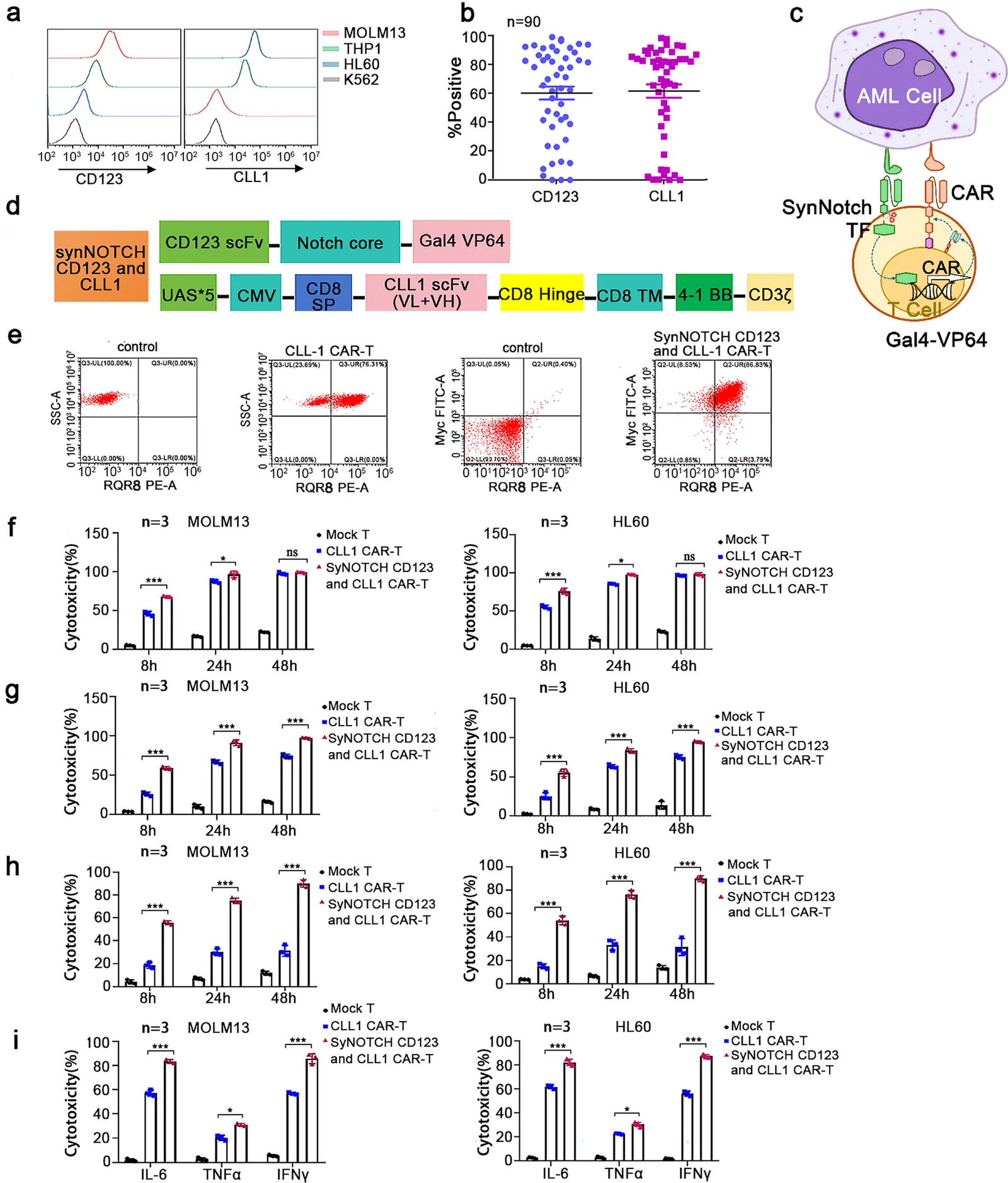
Screening of SynNotch “AND” logical gating targets to mitigate the off-tumor effects of CLL1 CAR-T cells. **a** Flow cytometry analysis of CD123 and CLL1 surface expression on AML cell lines (MOLM13, THP1, HL60) and the CML cell line K562. **b** Scatter plot summarizing the expression levels of CD123 and CLL1 in primary leukemic blasts from 90 patients diagnosed with AML. **c** Schematic illustration of the SynNotch “AND” logic-gated T-cell recognition system targeting AML cells. **d** Schematic representation of the lentiviral vector construct encoding the SynNotch CD123 receptor and CLL1 CAR tandem genes. **e** Representative flow cytometry plots showing the transduction efficiency of Mock T cells, conventional CLL1 CAR-T cells, and SynNotch CD123 and CLL1 CAR-T cells. **f**–**h** Comparative cytotoxicity of Mock T cells, CLL1 CAR-T cells, and SynNotch CD123 and CLL1 CAR-T cells against MOLM13 and HL60 target cells at 8 h, 24 h, and 48 h over three sequential rounds of co-culture. **i** In the third killing experiment, the levels of TNF-α, IL-6 and IFN-γ, produced during co-culture of MOLM13 and HL60 cells with Mock T cells, CLL1 CAR-T cells, and SynNotch CD123 and CLL1 CAR-T cells were compared. Results are expressed as mean ± SD from three independent experiments (*n* = 3). Statistical differences were evaluated using two-way ANOVA followed by multiple-comparison testing. *p < 0.05, **p < 0.01, ***p < 0.001

Following exposure to the CD123 antigen, CLL1 CAR induction was detectable within 6 h, and its expression persisted for at least 48 h, preceding the complete elimination of the recognized tumor cells. The therapeutic efficacy and target specificity of the CD123-CLL1 SynNotch CAR-T system for AML were then evaluated. Flow cytometry was used to assess CD123 and CLL1 expression in AML cell lines (MOLM13, THP1, and HL60) and the CML cell line K562 (Fig. 2a). To assess whether the SynNotch design could reduce severe granulocytopenia associated with CLL1 CAR-T cells, patient samples were analyzed by flow cytometry. The analysis of CD123 and CLL1 expression in primary AML samples is shown in Fig. 2b and Table S1. Among the analyzed patients, 91% were positive for CD123, with a median expression level of 61%, whereas CLL1 was detected in 86% of patients, with a median expression level of 63% (range, 0%–100%). Co-expression of CD123 and CLL1 was observed in 80% of AML cases (Fig. 2b). A schematic diagram of SynNotch “AND” logic gating is shown in Fig. 2c. CLL1 single-target CAR-T cells and SynNotch CD123 and CLL1 CAR-T cells were generated using a lentiviral delivery system (Fig. 2d). The results showed successful introduction of the CLL1 CAR and SynNotch constructs into T cells, with transduction efficiencies of 76.31% and 86.83%, respectively (Fig. 2e). To evaluate the precise and persistent cytotoxicity of CLL1 CAR-T and SynNotch CD123 and CLL1 CAR-T cells against AML cell lines in vitro, CLL1- and CD123-expressing AML cell lines were co-cultured with CAR-T cells or untransduced T cells at an effector-to-target cell ratio (E:T) of 1:3. Figure 2f-h show the killing curves for the first, second, and third rounds at three time points, respectively. The experimental results indicated that SynNotch CD123 and CLL1 CAR-T cells maintained higher cytotoxic activity than CLL1 CAR-T cells throughout the three rounds of tumor challenge. These findings suggest that the SynNotch CD123 and CLL1 CAR-T cells constructed in this study had sustained cytotoxic activity. In addition, in the third round of the killing assay, compared with CLL1 CAR-T cells, SynNotch CD123 and CLL1 CAR-T cells produced significantly higher interferon-gamma (IFN-γ), IL-6, and tumor necrosis factor-alpha (TNF-α) levels against MOLM13 and HL60 cells at an E:T ratio of 1:3 (Fig. 2i). These results suggest that, compared with CLL1 CAR-T cells, SynNotch CAR-T cells showed stronger recognition and cytotoxicity toward AML cells, which may contribute to improved anti-AML activity. Moreover, SynNotch CAR-T cells exhibited more sustained anti-tumor activity and retained the ability to eliminate tumor cells over repeated challenges.

### SynNotch CD123 and CLL1 CAR-T cells can effectively lyse primary AML cells

**Fig. 3:**
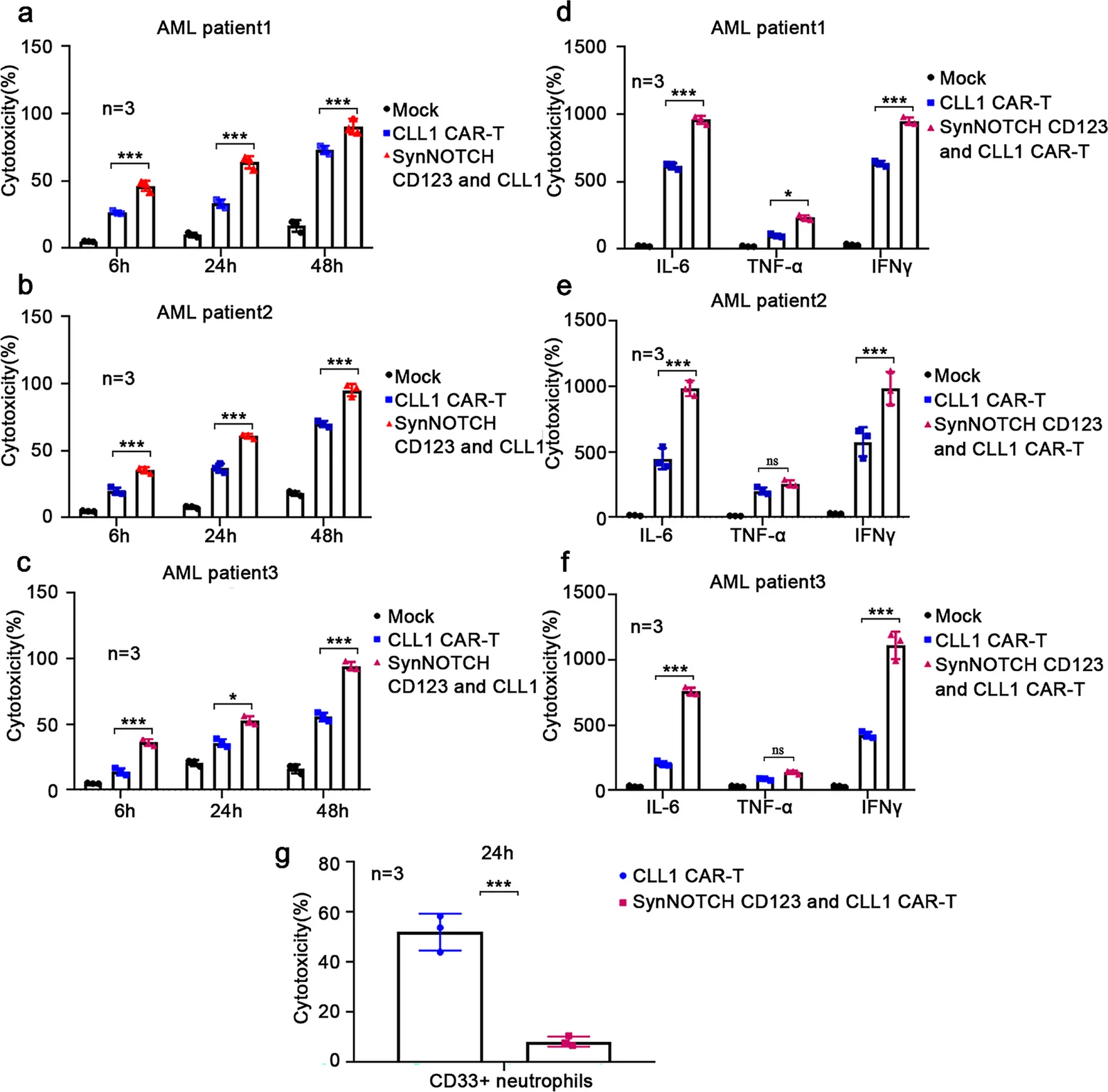
SynNotch CD123 and CLL1 CAR-T cells effectively lyse primary AML cells while reducing off-tumor toxicity against neutrophils. **a**-**c** Comparative cytotoxicity of Mock T cells, conventional CLL1 CAR-T cells, and SynNotch CD123 and CLL1 CAR-T cells against primary AML blasts isolated from three independent patients at 6 h, 24 h, and 48 h (*n* = 3). **d**-**f** Quantification of pro-inflammatory cytokines (IL-6, TNF-α, and IFN-γ) released in the co-culture supernatants after effector T cells were incubated with primary AML blasts from the indicated three patients (*n* = 3). **g** Evaluation of off-tumor cytotoxicity of CLL1 CAR-T and SynNotch CD123 and CLL1 CAR-T cells against normal CD33^+^ neutrophils harvested from healthy donors at 24 h of co-culture. Results are expressed as mean ± SD from three independent experiments (*n* = 3). Statistical differences were evaluated using two-way ANOVA followed by multiple-comparison testing. *p < 0.05, **p < 0.01, ***p < 0.001

To further assess the cytotoxicity of SynNotch CD123 and CLL1 CAR-T cells compared with CLL1 CAR-T cells, an in vitro cytotoxicity assay was conducted using primary AML cells isolated from patients with relapsed/refractory AML. Three primary AML samples obtained from peripheral blood (*n* = 3) were analyzed to determine CD123 and CLL1 expression levels. The clinical characteristics of these patients, including age, disease status, and baseline CD123/CLL1 co-expression profiles, are summarized in Table S2. Briefly, all three patients were diagnosed with relapsed/refractory AML and showed high co-expression of CD123 and CLL1 on primary blasts (range, 65%–85% for CD123 and 55%–78% for CLL1). To evaluate cell-killing efficiency, primary AML blasts were co-cultured with three groups of effector cells: (1) untransduced Mock T cells (negative control), (2) conventional CLL1 CAR-T cells (single-target control), and (3) SynNotch CD123 and CLL1 CAR-T cells (dual-target experimental group). The co-culture was performed in RPMI-1640 medium supplemented with 20% Fetal Bovine Serum (FBS) at an effector-to-target (E:T) ratio of 1:3. After 8, 24, and 48 h of incubation, target cell lysis was quantified by flow cytometry, and culture supernatants were harvested for cytokine profiling. As shown in Fig. 3a-c, SynNotch CD123 and CLL1 CAR-T cells exhibited significantly higher efficacy than CLL1 CAR-T cells against patient-derived primary AML cells. Moreover, co-culture of SynNotch CD123 and CLL1 CAR-T cells with primary AML cells resulted in markedly higher IL-6 and IFN-γ levels in the supernatants at 48 h (Fig. 3d-f). In addition, CD33-positive cells isolated from peripheral blood mononuclear cells (PBMCs) of healthy donors, representing neutrophil populations, were used to evaluate CAR-T cell-mediated cytotoxicity after 24 h of co-culture. The results demonstrated significantly reduced cytotoxicity of SynNotch CD123 and CLL1 CAR-T cells toward neutrophils (Fig. 3g). In summary, these findings indicate that SynNotch CD123 and CLL1 CAR-T cells retain potent anti-AML activity against primary AML cells while reducing neutrophil cytotoxicity. Therefore, the SynNotch “AND” CAR-T cell design may improve the targeting specificity of CLL1 CAR-T therapy and mitigate on-target, off-tumor toxicity.

### SynNotch CD123 and CLL1 CAR-T cells exhibit sustained activation

**Fig. 4:**
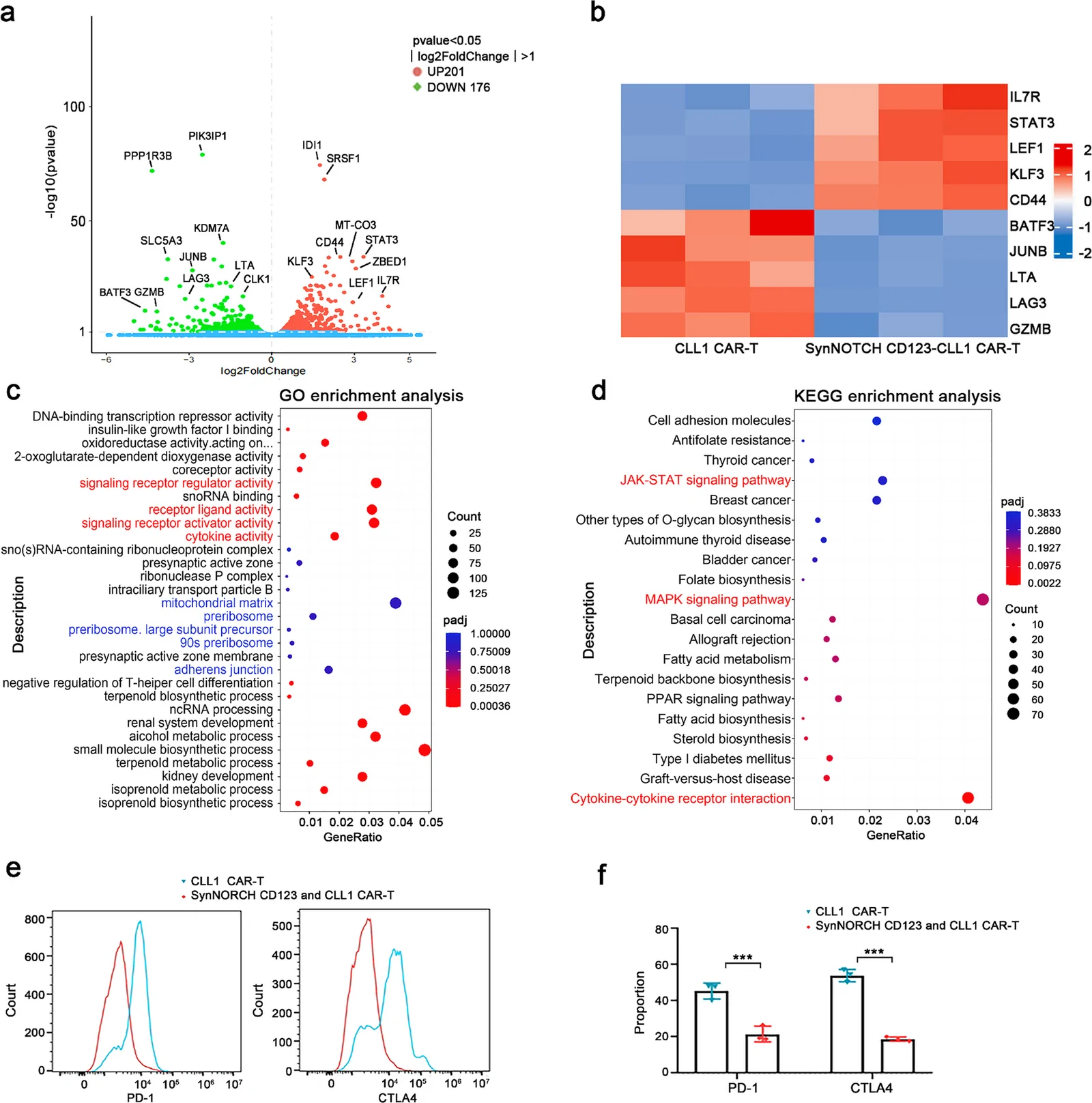
SynNotch CD123 and CLL1 CAR-T cells can cause sustained activation. **a** Volcano plot of transcriptome sequencing analysis showing the differentially expressed genes between SynNotch CD123 and CLL1 CAR-T cells and CLL1 CAR-T cells. Red and green dots denote upregulated (*n* = 201) and downregulated (*n* = 176) genes, respectively (|log2FoldChange|> 1, *P*-value < 0.05). **b** Heatmap depicting the expression levels of selected genes associated with T-cell exhaustion (LTA, BATF3, GZMB, LAG3, JUNB) and naïve/memory differentiation (IL7R, LEF1, CD44, KLF3) across the two groups. **c** Gene Ontology (GO) enrichment analysis highlighting the major biological processes associated with differentially expressed genes. **d **Kyoto Encyclopedia of Genes and Genomes (KEGG) pathway analysis identifying the principal signaling pathways enriched among the differentially expressed genes. **e** Representative flow cytometry histograms illustrating PD-1 and CTLA4 surface expression on SynNotch CD123 and CLL1 CAR-T cells and CLL1 CAR-T cells. **f** Quantitative analysis of PD-1 and CTLA4 surface expression in the indicated CAR-T groups. Results are expressed as mean ± SD from three independent experiments (*n* = 3). Statistical differences were evaluated using two-way ANOVA followed by multiple-comparison testing. *p < 0.05, **p < 0.01, ***p < 0.001

To identify transcriptomic differences underlying the functional divergence between conventional CLL1 CAR-T cells and SynNotch CD123 and CLL1 CAR-T cells, RNA-sequencing (RNA-Seq) was performed to compare the global gene expression profiles of the two CAR-T products. A volcano plot was generated to visualize the distribution of differentially expressed genes between the CLL1 CAR-T and SynNotch CD123 and CLL1 CAR-T cell groups (|log2FC|> 2, padj < 0.05; Fig. 4a). Genes downregulated in SynNotch CD123 and CLL1 CAR-T cells included exhaustion-associated genes such as lymphotoxin alpha (LTA), basic leucine zipper ATF-like transcription factor 3 (BATF3), granzyme B (GZMB), lymphocyte activating 3 (LAG3), and JunB proto-oncogene, AP-1 transcription factor subunit (JUNB). Upregulated genes included those associated with naïve and memory differentiation, such as interleukin 7 receptor (IL7R), lymphoid enhancer binding factor 1 (LEF1), CD44 molecule (CD44), and Kruppel like factor 3 (KLF3) (Fig. 4b). In SynNotch CD123 and CLL1 CAR-T cells, other significantly downregulated genes included protein phosphatase 1 regulatory subunit 3B (PPP1R3B), phosphoinositide-3-kinase interacting protein 1 (PIK3IP1), solute carrier family 5 member 3 (SLC5A3), and lysine demethylase 7 A (KDM7A), whereas other upregulated genes included isopentenyl-diphosphate delta isomerase 1 (IDI1), serine and arginine rich splicing factor 1 (SRSF1), mitochondrially encoded cytochrome c oxidase III (MT‑CO3), signal transducer and activator of transcription 3 (STAT3), and zinc finger BED-type containing 1 (ZBED1). Functional enrichment analyses were performed to explore potential mechanisms. As shown in Fig. 4c, Gene Ontology (GO) analysis showed that upregulated differentially expressed genes (DEGs) were mainly related to the immune response and cytokine-mediated signaling pathways, whereas downregulated DEGs were enriched in metabolic and redox processes. Kyoto Encyclopedia of Genes and Genomes (KEGG) analysis showed that the mitogen-activated protein kinase (MAPK) signaling pathway and Janus kinase-signal transducer and activator of transcription (JAK-STAT) signaling pathway were among the most enriched pathways in the upregulated genes (Fig. 4d), suggesting that altered inflammatory signaling cascades may contribute to these changes. The downregulated genes were related to immune dysfunction pathways. Figure 4e, f show that the expression levels of the immune checkpoint molecules programmed cell death protein 1 (PD-1) and cytotoxic T-lymphocyte-associated protein 4 (CTLA4) in SynNotch CD123 and CLL1 CAR-T cells were lower than those in the CLL1 CAR-T cell group, further supporting the transcriptomic findings at the protein level. These results indicate that genes downregulated in SynNotch CD123 and CLL1 CAR-T cells overlapped with exhaustion-associated genes, while genes upregulated in SynNotch CD123 and CLL1 CAR-T cells overlapped with genes associated with naïve/memory differentiation.

### SynNotch CD123 and CLL1 CAR-T cells demonstrate effective clearance of leukemic cells in a xenograft model of AML

**Fig. 5:**
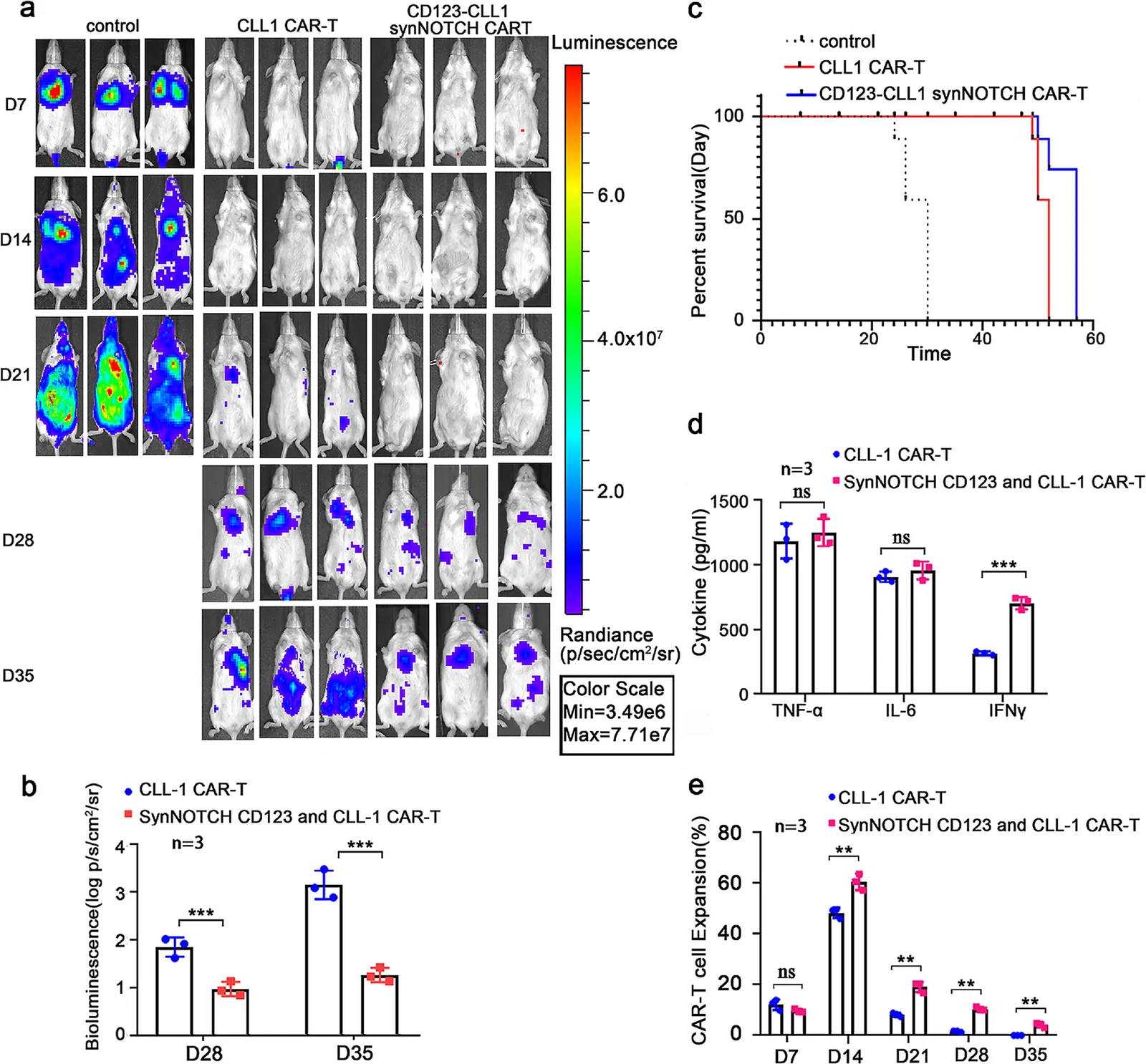
SynNotch CD123 and CLL1 CAR-T cell treatment was associated with a marked reduction in leukemic burden in the AML xenograft model. **a** Representative bioluminescence imaging (BLI) showing the tumor burden in AML xenograft mice treated with Mock T cells, CLL1 CAR-T cells, and SynNotch CD123 and CLL1 CAR-T cells at the indicated time points. **b** Quantification of tumor progression, depicted as the total bioluminescence flux (photons/s) over time in the indicated treatment groups. **c** Kaplan–Meier survival curves comparing among different groups of mice. Survival differences between groups were assessed using the log-rank test, with corresponding *P* values shown in the graph. **d** Cytokine concentrations in peripheral blood collected on day 14 post-infusion. **e** Percentage of CAR-T cell expansion among CD3-positive T cells in peripheral blood at different time points. Results are expressed as mean ± SD from three independent experiments (*n* = 3). Statistical differences were evaluated using two-way ANOVA followed by multiple-comparison testing. *p < 0.05, **p < 0.01, ***p < 0.001

To assess the in vivo antileukemic efficacy of SynNotch CD123 and CLL1 CAR-T cells in comparison with CLL1 CAR-T cells, AML xenograft models were established in NSG mice. On day 0, 2 × 10^6^ MOLM-13 cells were intravenously injected into mice, followed by injection of 2.5 × 10^6^ SynNotch CD123 and CLL1 CAR-T or CLL1 CAR-T cells on day 3 after tumor inoculation. Leukemia progression was monitored weekly using bioluminescence imaging (BLI). As shown in Fig. 5a, the longitudinal changes in tumor burden among the different treatment groups were assessed over the indicated time points. Mice treated with either CLL1 CAR-T or the dual-targeting SynNotch construct demonstrated a marked suppression of tumor expansion relative to the control cohort receiving untransduced T cells. Quantification of total bioluminescence flux confirmed the changes in tumor burden (Fig. 5b). Consistent with the reduction in tumor burden, mice treated with either CLL1 CAR-T cells or SynNotch CD123 and CLL1 CAR-T cells exhibited significantly improved survival compared with control groups (Fig. 5c). In addition, cytokine analysis showed higher IFN-γ release on day 14 in the SynNotch CD123 and CLL1 CAR-T cell treatment group compared with the CLL1 CAR-T cell group. TNF-α and IL-6 levels did not show a significant increase in the SynNotch CD123 and CLL1 CAR-T cell group (Fig. 5d). SynNotch CD123 and CLL1 CAR-T cells were monitored longitudinally in vivo by flow cytometry using the Myc tag for the SynNotch receptor and RQR8 for CLL1 CAR as specific identification markers. The percentage of CAR-T cells among CD3^+^ T cells was measured serially to assess cell expansion and persistence (Fig. 5e). These results indicate that SynNotch CD123 and CLL1 CAR-T cells can recognize and eliminate AML cells, with greater in vivo persistence and improved therapeutic efficacy in this model. These findings support further evaluation of this strategy for relapsed/refractory AML.

## Discussion

The treatment response in patients with AML is highly variable because of the heterogeneous and complex nature of the disease [12, 13]. Characterizing the molecular and phenotypic profiles of AML is essential for providing patients with more effective treatment options. In recent years, several investigations have highlighted the promise of CAR-T therapies targeting canonical antigens, such as CD123, CD33 and CLL1, which are expressed relatively homogeneously and specifically on the surface of leukemia blasts [14–17]. However, clinical trials have shown that limited therapeutic efficacy and treatment-related toxicities remain major barriers to the broader clinical application of these CAR-T products [18].

As an innovative form of CAR-T cell therapy, SynNotch CAR-T technology can enhance specific tumor-cell recognition and killing by integrating synthetic Notch receptors with CAR-T cells. Compared with conventional CAR-T-cell therapies, SynNotch CAR-T technology offers several distinct advantages. First, the SynNotch receptor can be engineered to enable context-dependent CAR-T cell activation within the tumor microenvironment, thereby improving tumor specificity while limiting recognition and destruction of normal tissues. This strategy may reduce off-tumor toxicity and enhance the safety profile of CAR-T cell therapy [6]. Second, after recognizing tumor cells, SynNotch CAR-T cells can release specific cytokines through synthetic signaling pathways, further enhancing their anti-tumor activity. This mechanism may increase the cytotoxic efficiency of CAR-T cells and promote their persistence within the tumor microenvironment, leading to more durable therapeutic effects [19]. In addition, tumor cells can evade CAR-T cell recognition through alterations in the expression or presentation of surface antigens. However, SynNotch CAR-T technology can be programmed for multi-antigen recognition, which may help prevent immune evasion [20]. SynNotch CAR-T technology also exhibits high programmability, allowing flexible design and adjustment of CAR-T cell targeting strategies according to different tumor types and patient-specific characteristics [21]. This programmability may support more precise and individualized therapeutic regimens [6]. CAR-T cell therapy is frequently associated with cytokine release syndrome (CRS), a potentially life-threatening treatment-related complication, primarily resulting from excessive cytokine production following CAR-T cell activation. The SynNotch CAR-T platform enables more precise control of CAR-T cell activation through an antigen-dependent “AND” gating mechanism, which may help restrict uncontrolled cytokine release and reduce CRS severity [22]. In our in vivo animal experiments, cytokine analysis showed increased IFN-γ release on day 14 in the SynNotch CD123 and CLL1 CAR-T cell treatment group compared with the CLL1 CAR-T cell group. TNF-α and IL-6 levels in the SynNotch CD123 and CLL1 CAR-T cell group were not significantly increased (Fig. 5d). These findings suggest that SynNotch CD123 and CLL1 CAR-T cells may enhance tumor-killing capacity in AML treatment without significantly elevating IL-6 inflammatory cytokine levels. However, the increased IL-6 observed in vitro also indicates that excessive cytokine secretion may carry a risk of CRS, and controlled T-cell activation should be considered in subsequent clinical designs. Cytokine levels, including IFN-γ, TNF-α, and IL-6, are often interpreted as markers of enhanced T-cell function, but their relationship with the therapeutic index (the balance between efficacy and toxicity) is complex, particularly the association of IL-6 with the risk of CRS. These findings highlight the importance of temporal dynamics, and future studies should incorporate monitoring at multiple time points to better assess relationships between cellular function, efficacy, and toxicity. Accordingly, cytokine patterns associated with CRS should be interpreted separately from other toxicities, such as neurotoxicity and respiratory failure. Therefore, while the elevated TNF-α and IFN-γ observed in the SynNotch group indeed indicate stronger T cell activation, their clinical significance should be interpreted in conjunction with specific toxicity endpoints. Future studies should examine the temporal relationships between these cytokine changes and clinically meaningful toxicity endpoints, such as neurotoxicity (ICANS) and respiratory failure. This approach may enable a more comprehensive evaluation of the therapeutic index by balancing predictable toxicity risks associated with specific activation patterns against anti-tumor efficacy (e.g., high IFN-γ/TNF-α).

In this study, the novel CAR receptor was successfully coupled with the SynNotch receptor protein, facilitating programmable and targeted delivery to multiple antigens. The results showed that SynNotch CD123 and CLL1 CAR-T cells exhibited superior recognition and cytotoxicity toward AML cell lines and primary AML blasts compared with conventional CLL1 CAR-T cells in vitro [6]. Furthermore, in AML xenograft models, SynNotch CD123 and CLL1 CAR-T cells demonstrated greater persistence and prolonged survival [20]. We systematically evaluated the anti-leukemic activity and safety of SynNotch-based dual-target CD123 and CLL1 CAR-T cells in AML. Compared with conventional single-target CLL1 CAR-T cells, the SynNotch CAR-T system activates CLL1 CAR expression only after recognition of both CD123 and CLL1 antigens, thereby achieving more specific killing of AML cells while reducing damage to CLL1 single-positive normal neutrophils. This “AND” logic gating mechanism improves the targeting precision of CAR-T therapy and may a promising strategy to mitigate on-target, off-tumor toxicity, particularly severe neutropenia, which limits the clinical application of CLL1 CAR-T therapy. This strategy may contribute to enhanced disease control with an improved safety profile and may offer a potential strategy to overcome key limitations of current AML therapies.

Previous studies have demonstrated that CLL1 CAR-T therapy possesses significant anti-leukemic efficacy in patients with AML but is accompanied by high rates of neutropenia and infection [22]. Although CD123 CAR-T also shows some efficacy, it similarly exerts toxicity against normal myeloid progenitors. In recent years, the SynNotch receptor system has been used to construct logic-gated CAR-T cells in solid tumors [23], but it has not been systematically validated in AML. This study is the first to apply SynNotch technology to a dual-target CD123 and CLL1 CAR-T design in AML, providing comprehensive evidence from in vitro assays, transcriptomic analysis, and animal models. Unlike conventional dual-target co-expression CAR-T designs, this system achieves hierarchical “recognition-activation-execution” regulation, further reducing the risk of non-specific activation. The results demonstrate that SynNotch CD123 and CLL1 CAR-T not only preserves but may also enhance anti-AML activity while significantly reducing cytotoxicity against normal neutrophils and potentially lowering the risk of infectious complications. Given that patients with AML, especially elderly or relapsed/refractory cases, often face severe infection risks, this strategy has clear translational value. Moreover, transcriptomic analysis revealed that this CAR-T variant downregulated multiple T cell exhaustion-associated genes, such as LAG3, GZMB, and BATF3, and upregulated naïve/memory T cell-associated genes, such as IL7R, LEF1, and CD44, suggesting the potential for longer in vivo persistence and improved anti-tumor capacity. These findings provide a theoretical basis for future clinical trials.

Despite accumulating sufficient preclinical evidence proving its substantial translational potential, multiple critical and complex key issues remain to be thoroughly resolved prior to formal clinical trial launch. Future research will first aim to overcome the inherent defects of immunodeficient NSG xenograft models by adopting CD34⁺hematopoietic stem cell humanized NS mice to accurately reconstruct a humanized bone marrow microenvironment. This advanced model will enable long-term monitoring of off-target damage to normal hematopoietic cells and facilitate a comprehensive evaluation of potential chronic toxic risks, providing a more physiologically relevant safety profile. Furthermore, multi-center patient samples covering various genetic and molecular subtypes of AML will be systematically collected to further validate the universal and robust therapeutic performance of this dual-antigen targeting strategy. Relevant mechanistic experiments will also be carried out to clarify whether the observed discrepancy in CD123 and CLL1 co-expression abundance among different AML genotypes directly affects the ultimate killing capacity and efficacy of the engineered CAR-T cells. Additionally, sophisticated long-term in vivo cell tracing combined with repeated tumor rechallenge assays will be conducted to systematically validate the stable persistence and sustained functional activity of memory-biased SynNotch CAR-T cells in vivo, assessing their potential for long-term disease control.

Further refinement of the genetic vector architecture and SynNotch regulatory circuits is also deemed essential for successful clinical transformation. To enhance safety, inducible suicide gene elements and cytokine regulatory modules will be integrated into the vector backbone to effectively restrain excessive CAR-T activation and reduce the incidence of severe cytokine release syndrome, a common and dose-limiting toxicity. Researchers will also dedicate efforts to optimize the induction efficiency of the Gal4-UAS system and precisely tune the binding affinity of the anti-CD123 single-chain antibody. These optimizations are crucial to precisely regulate the activation threshold of the SynNotch receptor, so as to effectively cut down basal leakage expression of CAR in normal tissues that merely express low levels of CD123, thereby minimizing on-target/off-tumor effects. Beyond conventional lentiviral transduction, alternative non-viral delivery systems such as transposon-based methods will be actively explored to eliminate the hidden danger of random genomic insertion and further elevate the overall biosafety profile of the final cell therapy products.

In terms of developing combinatorial therapeutic regimens, this dual-gated SynNotch CAR-T platform will be strategically combined with immune checkpoint inhibitors, metabolic regulators, and epigenetic-modifying agents. The goal is to synergistically reverse the profoundly immunosuppressive bone marrow niche characteristic of AML and proactively suppress tumor relapse potentially triggered by antigen loss or downregulation. Benefiting from the highly modular and adaptable design of this AND-gate framework, the simple replacement of the antigen-targeted single-chain antibodies allows the platform to be readily generalized to target other myeloid neoplasms, such as myelodysplastic syndromes or high-risk myeloproliferative neoplasms. This versatility positions the platform as a universal and powerful template for developing next-generation precise immunotherapies against a broad spectrum of hematological malignancies.

Clinically, the present work provides solid and compelling preclinical support for initiating investigator-sponsored phase I trials targeting relapsed/refractory AML patients, particularly those at high risk of life-threatening neutropenia. Subsequent translational work will necessarily focus on establishing standardized GMP-grade CAR-T preparation processes, establishing a complete panel of quality release indicators, and meticulously designing graded dose-escalation clinical schemes to determine the optimal therapeutic window. Collectively, the CD123/CLL1 SynNotch AND-gate CAR-T represents a truly innovative precision immunotherapeutic modality that successfully decouples potent anti-leukemic effects from debilitating myeloid toxicity. Different antigens can be programmatically recognized and targeted by modulating the covalent assembly structure of CAR-T cells. Moreover, by adjusting the activation level of the CAR-SynNotch receptor, the specificity and activity of CAR-T cells can be precisely regulated. This construct enables CAR-T cells to recognize specific tumor antigens and activate downstream signaling pathways, thereby releasing anti-tumor effector molecules and auxiliary cell proliferation factors. Continuous mechanistic exploration and relentless technical optimization will undoubtedly accelerate its clinical translation, ultimately delivering safer and more durable long-lasting disease remission to AML patients who currently have severely limited available therapeutic options.

## Materials and methods

### Clinical cohort and data acquisition

Clinical data were collected from 59 patients with AML enrolled in a CLL1 CAR-T cell therapy trial were retrospectively analyzed. The study protocol was reviewed and received approval from the Institutional Review Board of Tianjin First Central Hospital. (2020N212KY). Clinical trial number: ChiCTR2000041054. Peripheral blood cell counts, cytokine profiles, and routine laboratory parameters were recorded at multiple time points, including before lymphodepleting treatment and within the first 14 days following CAR-T-cell infusion. Major adverse events were also recorded.

Inclusion criteria:

1. Eligible patients had relapsed/refractory acute myeloid leukemia (AML), with refractory disease defined according to the following criteria:
  1. Failure to achieve partial remission after 2 courses of standard remission-induction therapy;
  2. Relapse within 6 months after the first remission (early relapse);
  3. Relapse more than 6 months after the first remission, with failure to respond to retreatment using the original remission-induction regimen;
  4. Multiple relapses. Relapse was defined as the presence of > 5% leukemic cells in the bone marrow following complete remission (intramedullary relapse) or the development of leukemic involvement outside the bone marrow (extramedullary relapse), most commonly affecting the central nervous system or testes.
2. Leukemic cells were confirmed to express CLL1/CD33/CD123/NKG2D/LILRB2 (CD85d)/LILRB4 (CD85k)/ADGRE2/CCR1 targets;
3. KPS score > 60;
4. Expected survival time > 3 months;
5. Age 2–75 years, with no sex restriction;
6. Clinical diagnosis of high-risk, relapsed, or refractory disease, or inability to tolerate or receive standard-of-care treatment;
7. Absence of severe psychiatric or other mental health disorders.
8. Adequate cardiac, hepatic, renal, and pulmonary function, defined as follows: (a) hepatic function, ALT and AST < 3 × the upper limit of normal (ULN) and total bilirubin ≤ 34.2 μmol/L; (b) renal function, serum creatinine < 220 μmol/L; (c) pulmonary function, resting oxygen saturation ≥ 95%; and (d) cardiac function, left ventricular ejection fraction (LVEF) ≥ 40%.
9. No other serious diseases incompatible with this protocol (such as autoimmune diseases, immunodeficiency, organ transplantation);
10. Ability to comply with trial management and follow-up;
11. Voluntary participation in the study and provision of written informed consent.

Exclusion criteria:

1. A history of other malignancies;
2. Uncontrolled active infection;
3. Underlying conditions requiring systemic glucocorticoid therapy.
4. Acute and chronic GVHD;
5. T-cell inhibitor therapy;
6. Pregnancy or lactation;
7. Active hepatitis B;
8. Any other condition considered by the investigators to be incompatible with study participation, such as HIV infection or intravenous drug use, or conditions that could potentially confound the analysis of study outcomes.

### Primary cell isolation and cell lines

The study utilized the 293 T, K562, THP1, MOLM13, and HL60 cell lines, all procured from the American Type Culture Collection (ATCC). All cell lines were accompanied by original certificates of identity. Throughout the study, we routinely monitored cell morphology, growth kinetics, and doubling times, which remained consistent with the known characteristics of these cell lines, indicating no cross-contamination or phenotypic drift.Mycoplasma contamination was assessed by PCR in all cell lines, with no contamination detected. T lymphocytes and peripheral blood mononuclear cells (PBMCs) were harvested from patients with a confirmed diagnosis of AML. All CAR-T cells were derived from these patient-sourced T cells. Sample collection was conducted following ethics committee approval, with written informed consent obtained from all participants.

### Preparation of SynNotch CD123 and CLL1 CAR-T cells

The CLL1 CAR construct was generated as previously described [22]. The CD123 SynNotch receptor was generated by fusing an anti-CD123 single-chain variable fragment (scFv) with the intracellular domain of the Notch core and the Gal4VP64 transcriptional activator. All SynNotch receptor constructs contained a Myc tag to enable detection of surface expression. The resulting constructs were cloned into the lentiviral vector pHRSIN. The modified vector pHRLSIN, containing the Gal4-inducible promoter, was used to drive the expression of CLL1 CAR containing an RQR8 tag. Lentiviruses carrying anti-CD123_SynNotch_Gal4VP64 and Gal4UAS_CLL1 CAR were produced using lentiviral packaging kits (GeneCopoeia) and used to transduce activated human T cells at an MOI of 5. Cells were maintained in culture for 10–14 days before being subjected to functional assays.

### Evaluation of CAR-T cell characteristics

Transduction efficiency of the CLL1 CAR and SynNotch CD123/CLL1 constructs in T cells was determined by flow cytometric analysis. The Myc tag was used to detect the anti-CD123 scFv SynNotch receptor. Additionally, the Gal4UAS_CLL1 CAR construct incorporated the compact marker/suicide gene RQR8, which includes epitopes for CD34 and CD20 antigens [22]. Consequently, transduction efficiency for Gal4UAS_CLL1 CAR was quantified by flow cytometry following staining with an anti-CD34 antibody recognizing the CD34 epitope within RQR8. The standalone CLL1 CAR also contained the RQR8 marker/suicide gene.

### Cytotoxicity of CAR-T cells against primary cells and AML cell lines

In vitro cytotoxicity was determined by co-culturing CAR-T cells with AML cell lines or primary AML blasts at an E:T ratio of 1:3. To assess the serial cytotoxicity of CAR-T cells, a repeated tumor-challenge assay was performed. Briefly, CAR-T cells were co-cultured with target cells at an E:T ratio of 1:3 for 48 h during the first round of stimulation. After incubation, residual target cells were removed, and fresh target cells were added to initiate the second and third rounds of co-culture under identical conditions. Cytotoxic efficiency was calculated as previously described [23].

### Cytokine release

Serum levels of the pro-inflammatory cytokines tumor necrosis factor-α (TNF-α), interleukin-6 (IL-6), and interferon-γ (IFN-γ) were assessed using a 10-plex flow cytometry-based cytokine detection kit (BIORIGIN, BNCBA001-48 T). The assay was performed as follows: (1) Fifty microliters of standards or diluted serum/plasma were added to the corresponding wells. (2) Fifty microliters of vortexed microspheres were added to each well, with periodic mixing to prevent settling. The total volume was 100 μL/well. (3) Plates were subjected to shaking at 450 rpm for 2 min and subsequently incubated at 37 °C for 30 min. (4) For the first magnetic separation, the plate was placed on a magnetic stand for 5 min, and the supernatant was removed by flicking and blotting without pipetting. (5) For washing, the plate was removed from the magnet, 200 μL of wash buffer was added, and magnetic separation was repeated for 5 min before removing the supernatant. (6) Each well was supplemented with 100 μL of detection antibody and incubated at 37 °C for 30 min. (7) The washing procedure described in steps 4–5 was repeated. (8) PE-conjugated streptavidin (100 μL/well) was then added, and the plate was incubated at 37 °C for 15 min. (9) The washing procedure described in steps 4–5 was repeated. (10) The samples were resuspended in 200 μL of wash buffer and subsequently analyzed by flow cytometry. At the end of each round, tumor cell lysis was quantified by flow cytometry, and supernatants were collected for measurement of IFN-γ, IL-6, and TNF-α. Mouse TNF-α ELISA Kit (Solarbio, SEKM-0034), mouse IL-6 ELISA Kit (Solarbio, SEKM-0007), and mouse IFN-γ ELISA Kit (Solarbio, SEKM-0031) were used for cytokine detection.

### Transcriptome sequencing (RNA-Seq)

On day 5 post-transfection, transcriptome sequencing was conducted on expanded CAR-T cells following 48 h of co-culture with tumor cells at an effector-to-target (E:T) ratio of 1:1. After two rounds of cytotoxicity assays, CAR-T cells expressing the RQR8 marker were isolated using CD34-conjugated magnetic beads. Isolated CAR-T cells were washed with ice-cold phosphate-buffered saline (PBS), collected by centrifugation, and immediately snap-frozen in liquid nitrogen before storage at −80 °C. Total RNA was subsequently extracted, and mRNA was purified using Oligo(dT) magnetic beads for subsequent library construction. Libraries were quantified using Qubit fluorometry and adjusted to a final concentration of 1.5 ng/µL. The distribution of insert sizes was assessed using an Agilent 2100 Bioanalyzer, and library concentrations were further determined by qRT-PCR before sample pooling and sequencing on an Illumina platform. Gene expression levels were calculated for each sample, and statistical analysis was performed to identify significantly differentially expressed genes.

### Evaluation of the efficacy and adverse effects of CAR-T cells in AML mouse xenograft models

Male NSG mice aged 6–8 weeks were obtained from Beijing Weidun Lihua Experimental Animal Technology Co., Ltd. and maintained under standard conditions in the animal facility at Tianjin Medical University. The AML xenograft model was established by intravenous injection of 1 × 10^6^ luciferase-expressing THP-1 cells. Three days after tumor cell inoculation, 2.5 × 10^6^ CAR-T cells or untransduced T cells were administered intravenously. At the indicated time points during the treatment period, tumor progression was monitored by bioluminescence imaging using an IVIS Lumina II system (PerkinElmer) after intraperitoneal administration of 3 mg D-luciferin (Sigma). Peripheral blood was collected via the tail vein, and blood cell counts were determined using an automated hematology analyzer. All animal studies were conducted in accordance with institutional guidelines and were approved by the Institutional Animal Care and Use Committee (No. 2025-SYDWLL-000096).

### Statistical analysis

GraphPad Prism 9.0 (GraphPad Software, San Diego, CA, USA) was used for statistical analysis. For data meeting the assumption of normality, results are expressed as mean ± standard deviation (SD), with paired *t*-tests used to compare two related groups and one-way analysis of variance (ANOVA) applied to comparisons involving multiple groups. Data that did not follow a normal distribution are reported as median values with the interquartile range (IQR) and were evaluated using the Kruskal–Wallis test. Survival outcomes were assessed by Kaplan–Meier analysis, with differences between survival curves determined using the log-rank test. Statistical significance was defined as a *P* value < 0.05.

## Supplementary Information

Supplementary Material 1.

## Data Availability

All data necessary for confirming the conclusions are included in this article. The transcriptomics data generated in this study has been deposited in the Science Data Bank (ScienceDB). The dataset is now publicly available with the following permanent identifiers: CSTR: https://cstr.cn/31253.11.sciencedb.010ak; DOI: https://doi.org/10.57760/sciencedb.010ak.
