# Supplementary material for: Synthetic Notch CD123 and CLL1 chimeric antigen receptor T-cells (CAR-T) target acute myeloid leukemia and reduce off-tumor effects associated with CLL1 CAR-T cells
Source: Mol Biomed. 2026 Oct 16;7:215. doi: 10.1186/s43556-026-00598-y (PMC13675449; doi:10.1186/s43556-026-00598-y)
Supplement: Supplementary file 1 [file 43556_2026_598_MOESM1_ESM.docx]

**Supplementary manuscript**

**Synthetic Notch CD123 and CLL1 chimeric antigen receptor T-cells (CAR-T)** **target acute myeloid leukemia and reduce off-tumor effects associated with CLL1 CAR-T cells**

Rui Zhang^1^, Na Chen^2^, Mengnan Li^3^, Qingxi Liu^4#^, Mingfeng Zhao^1#^

**Affiliation:**

1 Department of Hematology, Tianjin First Central Hospital, Tianjin, 300380, China
2 First Central Hospital of Tianjin Medical University, Tianjin Medical University, Tianjin, China
3 School of Medicine, Nankai University, Tianjin, China
4 Qilu Institute of Technology, Shandong, China

\# Corresponding Author: Qilu Institute of Technology, Shandong, China; [liuqingxi66@aliyun.com;](mailto:liuqingxi66@aliyun.com;) Mingfeng Zhao, Department of Hematology, Tianjin First Central Hospital, China; email: [mingfengzhao@sina.com](mailto:mingfengzhao@sina.com);

**Supplementary table 1:** Representative examples of CD123 and CLL1 expression on primary cells.

|  | **Sample Source** | **Sex** | **Age** | **Status** | **Subtype** | **WBC (*10^9^)** | **Blast (%)** | **Cytogenetics Abnormality** | **All Flow Results List** | **CD123 positive (%)** | **CLL1 positive (%)** |
| --- | --- | --- | --- | --- | --- | --- | --- | --- | --- | --- | --- |
| **1** | **BM** | **Female** | **73** | **New diagnosed** | **M4** | **206.39** | **51.72** | **FLT3-ITD; NPM1; DNMT3A** | **CD34+CD33+CD13+CD38+CD96+MPOdim+CD7-CD19-CD56-CD11b-CD14-CD64-CD11c-CD15-CD36-cCD7a-cCD3-** | **74.82** | **68.34** |
| **2** | **BM** | **Male** | **29** | **New diagnosed** | **M4** | **1.41** | **9.46** | **FLT3-TKD; FLT3-TID; CEBPA; TET2** | **CD117+CD34dim+CD33bri+HLA-DA+CD19-CD56-C14-CD96-CD11b-CD38-** | **43.28** | **83.39** |
| **3** | **BM** | **Male** | **54** | **New diagnosed** | **M2** | **7.15** | **22.00** | **MLL-AF9** | **CD117+CD33dim+HLA-DRdim+CD38+MPO+CD7-CD19-CD56-CD34-CD13-CD16-CD14-CD64-CD15-CD138-CD9-cCD7a-cCD3-** | **83.91** | **98.45** |
| **4** | **BM** | **Female** | **47** | **Relapse** | **M5** | **4.80** | **83.55** | **FLT3-TID** | **CD34+CD33bri+HLA-DR+CD38+CD14dim+CD7-CD19-CD56-CD15-** | **98.26** | **88.36** |
| **5** | **BM** | **Female** | **73** | **Relapse** | **M5** | **4.12** | **65.46** | **DNMT3A; IDH2** | **CD117+CD34+CD33+CD7-CD19-CD56-CD15-CD96-CD11b-CD14-** | **92.61** | **82.92** |
| **6** | **BM** | **Male** | **43** | **New diagnosed** | **M5** | **13.90** | **23.24** | **TET2; FLT3-TKD; NPM1; NRAS** | **CD117+CD33bri+HLA-DR+CD13+CD96+MPOdim+CD7-CD19-CD56-CD34-CD16-CD14-CD64-CD15-CD36-cCD7a-cCD3-** | **90.01** | **84.78** |
| **7** | **BM** | **Female** | **79** | **New diagnosed** | **M5** | **11.49** | **32.29** | **EZH2; RUNX1; KRAS; DNMT3A; ASXL1; SRSF2; SH2B3** | **CD117dim+CD34+CD33+HLA-DR+CD13+CD10dim+CD38+CD300e+CD7-CD19-CD56-CD16-CD64-CD11b-CD14-CD36-CD15-MPO-cCD3-** | **63.23** | **80.78** |
| **8** | **BM** | **Male** | **76** | **Relapse** | **M5** | **82.43** | **84.22** | **NPM1; DNMT3A; TET2** | **CD117+CD34+CD33bri+HLA-DRdim+CD96+CD7-CD19-CD56-CD11b-CD14-CD15-** | **60.73** | **49.70** |
| **9** | **BM** | **Female** | **68** | **New diagnosed** | **M5** | **20.06** | **22.48** | **DNMT3A; EZH2; RUNX1; ASXL1; PHF6** | **CD117dim+CD34+CD33bri+HLA-DR+CD13+CD7-CD19-CD56-CD16-CD300e-CD11b-CD14-CD64-** | **18.23** | **98.35** |
| **10** | **BM** | **Male** | **45** | **New diagnosed** | **M4** | **2.42** | **70.00** | **CEBPA; RUNX1** | **CD117+CD33+CD34+ CD7-CD19-CD96-CD14-CD15-** | **85.55** | **80.50** |
| **11** | **BM** | **Female** | **67** | **New diagnosed** | **M5** | **72.27** | **5.78** | **FLT3-ITD; NPM1; DNMT3** | **CD117+CD33bri+HLA-DR+CD13+CD64+CD38+CD96+MPO+CD7-CD19-CD56-CD16-CD11b-CD14-CD300e-CD15-CD36-CD4-cCD79a-cCD3-** | **28.53** | **75.70** |
| **12** | **BM** | **Male** | **64** | **New diagnosed** | **M5** | **9.78** | **46.78** | **FLT3-TKD** | **CD117dim+CD34+HLA-DRdim+CD33+CD13+cCD79adim+CD7-CD19-CD56-CD16-CD14-CD38-CD64-CD11c-CD15-MPO-cCD3-** | **57.98** | **3.05** |
| **13** | **BM** | **Female** | **70** | **New diagnosed** | **M2** | **45.74** | **90.97** | **DNMT3A; IDH** | **CD117+CD34+HLA-DRdim+CD33+CD13+CD38+CD96+MPO+CD19-CD56-CD16-CD14-CD11b-CD36-CD64-CD11c-CD4-cCD79a-cCD3-** | **87.55** | **45.98** |
| **14** | **BM** | **Female** | **49** | **New diagnosed** | **M5** | **93.89** | **15.01** | **MLL-AF9; WT1; ZRSR2** | **CD117+CD33+CD64+CD13+CD64+CD38+MPO+CD7-CD19-CD56-CD34-HLA-DR-CD14-CD16-CD15-CD123-CD4-cCD3-cCD79a-** | **31.57** | **57.60** |
| **15** | **BM** | **Male** | **51** | **New diagnosed** | **M5** | **0.82** | **45.31** | **ASXL1; DNMT3A; IDH1; PHF6** | **CD34+CD117+CD33dim+CD7-CD19-HLA-DR-CD11b-CD14-** | **90.56** | **72.83** |
| **16** | **BM** | **Female** | **32** | **New diagnosed** | **M2** | **38.84** | **66.18** | **CEBPA** | **CD34+CD117+CD33+HLA-DR+CD7+CD13+CD38+CD96+MPO+CD19-CD56-CD16-CD11b-CD14-CD64-CD11c-CD15-CD36-CD4-CD10-CD9-cCD3-** | **55.52** | **84.98** |
| **17** | **BM** | **Male** | **68** | **New diagnosed** | **M4** | **87.37** | **85.06** | **FLT3-ITD; NPM1; CEBPA; TET2; EZH2; ASXL1; ERSR2** | **CD117+CD33+CD13+CD38+MPO+HLA-DR-CD34-CD7-CD19-CD16-CD11b-CD64-CD11c-CD15-CD36-CD4-CD96-cCD79a-cCD3-** | **97.12** | **96.64** |
| **18** | **BM** | **Male** | **31** | **Relapse** | **M5** | **5.88** | **48.12** | **EZH2; WT1** | **HLA-DR+CD33bri+CD7+ CD19-CD15-CD14-** | **22.08** | **7.21** |
| **19** | **BM** | **Female** | **63** | **Relapse** | **M4** | **4.98** | **7.00** | **NUP98-HoxA9** | **CD117+CD33+CD34+HLA-DR-CD19-CD56-CD7-CD15-CD96-CD14-CD11b-** | **79.42** | **80.73** |
| **20** | **BM** | **Female** | **35** | **New diagnosed** | **M5** | **15.49** | **31.53** | **NRAS; EZH2** | **CD34+CD33+HLA-DR+CD13+CD38+CD7-CD19-CD56-CD16-CD11b-CD14-CD300e-CD15-MPO-cCD79a-cCD3-** | **92.76** | **97.40** |
| **21** | **BM** | **Male** | **4%** | **Relapse** | **M5** | **11.12** | **66.42** | **CEBPA; DNMT3A; TET2** | **CD117+CD33+HLA-DR+CD34+CD96+CD15-CD7-CD19-CD56-CD14-CD11b-** | **41.32** | **91.61** |
| **22** | **BM** | **Female** | **33** | **New diagnosed** | **M2** | **3.08** | **65.23** | **ASXL1; WT1; AML1-ETO** | **CD34+CD117+CD33+HLA-DR+CD56+CD13+CD38+CD64dim+CD96+MPO+CD7-CD19-CD16-CD11b-CD15-CD4-CD36-cCD79a-cCD3-** | **80.95** | **99.24** |
| **23** | **BM** | **Male** | **32** | **New diagnosed** | **M4** | **34.20** | **72.55** | **FLT3; RUNX1; WT1** | **CD34+CD117+CD33+HLA-DR+CD96+CD38+CD7-CD19-CD56-CD14-CD15-CD11b-** | **96.64** | **96.92** |
| **24** | **BM** | **Female** | **30** | **New diagnosed** | **M3** | **2.05** | **91.58** | **FLT3; NRAS; WT1** | **CD117+CD13+CD33+CD38+CD9+CD64+cMPO+cCD79adim+HLA-DR-CD19-CD11b-CD15-CD7-CD34-CD16-CD11c-CD56-CD14-CD10-cCD3-** | **18.54** | **5.05** |
| **25** | **BM** | **Female** | **70** | **New diagnosed** | **M2** | **44.64** | **29.58** | **NPM1; DNMT3A** | **CD117+CD33briCD13+HLA-DR+MPO+CD19-CD56-CD16-CD11b-CD14-CD64-CD11c-CD15-CD38-cCD79a-cCD3-** | **7.08** | **18.08** |
| **26** | **BM** | **Male** | **49** | **New diagnosed** | **M3** | **4.91** | **91.80** | **-** | **CD117+CD13+CD33+CD38+CD9+CD64+cMPO+cCD79adim+CD96+HLA-DR-CD19-CD11b-CD15-CD7-CD34-CD16-CD11c-CD56-CD14-CD10-cCD3-** | **7.27** | **12.05** |
| **27** | **BM** | **Male** | **72** | **New diagnosed** | **M2** | **2.37** | **52.34** | **KIT; DNMT3A** | **CD34+CD117+HLA-DR+CD33+CD56+CD19+CD13+CD11c+CD38+CD96+cCD79a+CD7-CD16-CD11b-CD14-CD64-CD15-CD36-cMPO-cCD3-** | **11.23** | **52.89** |
| **28** | **BM** | **Female** | **82** | **New diagnosed** | **M5** | **98.36** | **62.72** | **FLT3; NPM1; DNMT3A; ASXL1** | **CD117+HLA-Drdim+CD34+CD33+CD13+CD38+CD96+MPO+CD7-CD19-CD56-CD16-CD11b-CD9-CD36-CD14-CD64-CD11c-CD15-cMPO-cCD3-** | **63.00** | **8.02** |
| **29** | **BM** | **Female** | **19** | **New diagnosed** | **M2** | **0.38** | **12.45** | **NRAS; PHF6; CCND2; PDGFRA; TTN; CSMD1; GATA1** | **CD34+CD33+HLA-DR+D117+CD7+CD38+CD19-CD56-CD14-CD15-CD96-CD11b-** | **78.01** | **82.92** |
| **30** | **BM** | **Male** | **52** | **New diagnosed** | **M2** | **3.29** | **19.36** | **AML1-ETO** | **CD34+CD117+CD56+CD33+HLA-DR+CD7-CD19-CD15-CD14-CD11b-CD96-CD38-** | **82.10** | **85.91** |
| **31** | **BM** | **Male** | **27** | **Relapse** | **M5** | **4.86** | **16.85** | **-** | **CD117+CD34+CD33+CD56+HLA-DRdim+CD33dim+CD13+CD38+CD7-CD19-CD15-CD14-CD11b-CD96-CD36-** | **56.28** | **87.05** |
| **32** | **BM** | **Male** | **47** | **Relapse** | **M4** | **3.28** | **86.50** | **RUNX1** | **CD117+HLA-DR+CD33bri+CD96+CD38+CD7-CD19-CD56-CD15-CD14-CD11b-** | **1.24** | **12.78** |
| **33** | **BM** | **Male** | **35** | **New diagnosed** | **M2** | **24.01** | **56.77** | **ASXL1; NRAS; TET2** | **CD117-CD34+HAL-DR+CD33+CD38+CD7-CD19-CD56-CD14-CD15-CD11b-** | **55.40** | **58.32** |
| **34** | **BM** | **Female** | **75** | **New diagnosed** | **M5** | **0.70** | **14.34** | **-** | **CD34+CD117+HLA-Drdim+CD13+CD64dim+CD96+CD7-CD19-CD56-CD16-CD14-CD123-** | **79.70** | **91.59** |
| **35** | **BM** | **Female** | **54** | **Relapse** | **M4** | **2.12** | **68.28** | **NARS** | **CD34bri+CD117+HLA-DR+CD33+CD7-CD19-CD56-CD14-CD11b-CD96-CD15-** | **64.69** | **52.78** |
| **36** | **BM** | **Female** | **55** | **New diagnosed** | **M5** | **351.47** | **87.36** | **-** | **CD33bri+HLA-DR+CD13+MPO+CD7-CD19-CD56-CD34-CD16-CD11b-CD14-CD11c-CD15-CD96-CD4-cCD79a-cCD3-** | **70.44** | **33.71** |
| **37** | **BM** | **Female** | **67** | **New diagnosed** | **M5** | **7.13** | **30.40** | **NRAS; FLT3-ITD; DNMT3A; TST2; NPM1** | **CD117+CD33bri+HLA-DR+CD19-CD56-CD14-CD11b-** | **97.67** | **83.98** |
| **38** | **BM** | **Male** | **54** | **Relapse** | **M5** | **0.88** | **33.67** | **WT1** | **CD117+CD34+HLA-DRdim+CD33+CD13+CD4dim+CD7-CD19-CD56-CD16-CD11b-CD14-CD64-CD300e-CD15-CD38-CD96-CD36-cCD79a-cCD3-** | **71.89** | **47.07** |
| **39** | **BM** | **Male** | **66** | **New diagnosed** | **M5** | **5.41** | **23.27** | **RUNX1** | **CD34+CD117+CD33+CD13+CD64dim+MPO+CD7-CD19-CD56-HLA-DR-CD16-CD11b-CD14-CD300e-CD15-CD38-CD36-CD1a-cCD79a-cCD3-** | **60.20** | **62.17** |
| **40** | **BM** | **Female** | **60** | **New diagnosed** | **M5** | **28.44** | **9.32** | **-** | **CD34+CD117+CD33bri+HLA-DR+CD13+CD38+CD7-CD19-CD56-CD16-CD11b-CD14-CD300e-CD15-** | **53.62** | **87.69** |
| **41** | **BM** | **Female** | **56** | **Relapse** | **M2** | **0.84** | **11.24** | **WT1** | **CD34+CD117+CD33+HLA-DR+CD19+CD7-CD56-** | **28.27** | **59.27** |
| **42** | **BM** | **Male** | **88** | **New diagnosed** | **M5** | **24.40** | **52.08** | **-** | **CD117+CD33+ CD7-CD19-CD56-CD34-HLA-DR-** | **1.75** | **97.08** |
| **43** | **BM** | **Female** | **18** | **Relapse** | **M2** | **0.13** | **20.41** | **FLT3-ITD; RUNX1** | **CD34+CD117+CD33bri+HLA-DR+CD7-CD19-CD56-CD15-CD14-CD11b-** | **88.70** | **73.25** |
| **44** | **BM** | **Female** | **70** | **New diagnosed** | **M5** | **90.04** | **96.42** | **-** | **CD117+CD33+CD38+CD64+CD13+MPO+CD34-CD7-CD19-HLA-DR-CD14-CD11c-CD16-CD11b-cCD79a-cCD3-** | **59.46** | **92.12** |
| **45** | **BM** | **Male** | **57** | **New diagnosed** | **M5** | **98.41** | **89.37** | **FLT3-ITD; WT1** | **CD117+HLA-DR+CD33bri+CD13+CD11cdim+CD38+MPO+cCD79adim+CD19-CD56-CD16-CD11b-CD14-CD64-CD15-CD36-CD10-CD4-CD2-CD24-cCD3-** | **95.40** | **68.87** |
| **46** | **BM** | **Female** | **15** | **New diagnosed** | **M5** | **0.55** | **81.58** | **WT1** | **CD117+CD33+CD13+CD64+CD9+CD38+MPO+CD7-CD19-CD56-CD34-HLA-DR-CD16-CD11b-CD14-CD11c-CD15-CD10-CD36-cCD79a-cCD3-** | **67.81** | **18.37** |
| **47** | **BM** | **Male** | **81** | **New diagnosed** | **M5** | **228.14** | **24.30** | **-** | **CD117+CD56+CD33bri+CD13+CD64dim+MPO+CD7-CD19-CD34-HLA-DR-CD16-CD11b-CD14-CD15-CD96-CD36-cCD79a-cCD3-** | **91.15** | **99.88** |
| **48** | **BM** | **Male** | **56** | **New diagnosed** | **M5** | **3.20** | **69.26** | **WT1** | **CD34+CD117+CD33+HLA-DRdim+CD7+CD13+CD5+CD38+CD4dim+CD3dim+ CD19-CD16-CD11b-CD14-CD64-CD15-CD36-CD96-cCD79a-CD2-CD3-CD8-** | **90.00** | **15.83** |
| **49** | **BM** | **Female** | **65** | **Relapse** | **M5** | **20.66** | **64.70** | **FLT3-ITD; DNMT3A; NPM1; TET2** | **CD38+CD33+CD117+CD34+ CD19-CD7-CD64-** | **98.13** | **99.52** |
| **50** | **BM** | **Male** | **57** | **New diagnosed** | **M5** | **8.41** | **81.86** | **MLL-ELL; NRAS** | **CD33bri+HLA-DR+CD64+CD38+MPO+CD7-CD19-CD56-CD34-CD13-CD16-CD14-CD300e-CD15-CD10-cCD3-cCD79a-** | **97.86** | **98.20** |
| **51** | **BM** | **Male** | **54** | **Relapse** | **M2** | **8.71** | **40.38** | **-** | **CD34+CD117+CD33+HLA-DR+CD7-CD19-CD56-CD15-CD14-CD96-CD11b-CD38-** | **66.59** | **11.21** |
| **52** | **BM** | **Male** | **72** | **New diagnosed** | **M5** | **2.56** | **94.08** | **DNMT3A; RUNX1** | **CD117+CD33+HLA-DRdim+CD7-CD19-CD56-CD14-CD11b-** | **51.22** | **99.96** |
| **53** | **BM** | **Male** | **44** | **Relapse** | **M3** | **1.34** | **83.23** | **PML-RARA** | **CD117+CD33bri+CD56+CD13+CD7-CD34-HLA-DR-CD16-CD11b-CD19-** | **94.03** | **79.32** |
| **54** | **BM** | **Female** | **48** | **Relapse** | **M5** | **8.61** | **8.67** | **-** | **CD33bri+CD56+HLA-DRdim+CD96+CD38+CD7-CD19-CD15-CD14-CD11b-** | **65.13** | **96.05** |
| **55** | **BM** | **Female** | **58** | **Relapse** | **M5** | **17.33** | **37.00** | **FLT3-ITD; NPM1; DNMT3A** | **CD117+CD96+CD33+HLA-DR+CD7+CD19-CD56-CD15-CD14-CD11b-** | **91.77** | **61.58** |
| **56** | **BM** | **Male** | **67** | **New diagnosed** | **M2** | **1,05** | **49.61** | **AML-ETO** | **CD117+CD34+HLA-DR+CD33+CD19+CD96+CD7-CD11b-CD15-CD14-** | **74.93** | **97.97** |
| **57** | **BM** | **Female** | **46** | **New diagnosed** | **M2** | **2.39** | **63.49** | **NPM1; DNMT3A; NRAS; WT1** | **CD117+HLA-DR+CD33bri+CD96+CD56-CD19-CD15-CD14-CD11b-** | **95.99** | **80.84** |
| **58** | **BM** | **Male** | **68** | **New diagnosed** | **M5** | **7.30** | **46.42** | **ASXL1; IDH2** | **CD34+CD117+HLA-DR+CD33+CD19-CD56-CD14-CD15-CD96-CD11b-CD38-** | **91.40** | **66.41** |
| **59** | **BM** | **Female** | **52** | **New diagnosed** | **M2** | **2.24** | **7.14** | **CEBPA** | **CD34+CD117+CD33bri+HLA-DR+CD7+CD96+CD38+CD19-CD56-CD14-CD15-CD11b-** | **52.40** | **40.93** |
| **60** | **BM** | **Male** | **42** | **New diagnosed** | **M1** | **5.28** | **42.43** | **-** | **CD117+CD33+HLA-DRdim+CD13+MPO+CD7-CD19-CD56-CD14-CD16-CD64-CD15-CD36-CD96-cCD79a-cCD3-** | **79.13** | **51.48** |
| **61** | **BM** | **Female** | **72** | **New diagnosed** | **M5** | **6.19** | **53.96** | **ASXL1; TET2; ETV6; JAK2; WT1** | **CD117+CD34+CD33+HLA-DR+CD13+CD96+MPO+CD19-CD16-CD56-CD14-CD64-CD11c-CD15-CD36-CD4-CD9-cCD79a-cCD3-** | **72.58** | **95.41** |
| **62** | **BM** | **Male** | **39** | **New diagnosed** | **M4** | **28.92** | **88.79** | **-** | **CD117+CD34+CD33+HLA-DR+CD13+CD96+MPO+CD19-CD16-CD56-CD14-CD64-CD11c-CD15-CD36-CD4-CD9-cCD79a-cCD3-** | **72.58** | **95.41** |
| **63** | **BM** | **Female** | **46** | **New diagnosed** | **M5** | **0.81** | **12.12** | **-** | **CD117+CD34+HLA-DR+CD33bri+CD7-CD19-CD56-CD15-CD96-CD14-CD11b-** | **86.48** | **76.16** |
| **64** | **BM** | **Female** | **16** | **New diagnosed** | **M4** | **16.76** | **75.69** | **CEBPA** | **CD117+CD34+CD33+HLA-DR+CD7+CD96+CD19-CD56-CD15-CD14-CD11b-** | **62.67** | **62.87** |
| **65** | **BM** | **Male** | **34** | **New diagnosed** | **M4** | **51.80** | **85.23** | **WT1** | **CD117+CD33+HLA-DR+CD13+MPO+CD7-CD19-CD56-CD11b-CD14-CD64-CD11c-CD15-CD36-CD4-cCD79a-cCD3-** | **92.65** | **64.77** |
| **66** | **BM** | **Female** | **45** | **New diagnosed** | **M4** | **2.37** | **20.07** | **WT1** | **CD34+CD117+HLA-DRdim+CD33+CD13+CD96+CD2dim+CD7-CD19-CD56-CD16-CD11b-CD14-CD11b-CD14-CD64-CD11c-CD15-CD38-CD36-CD96-CD4-CD7-CD3-CD8-CD5-cCD3-cCD79a-** | **63.25** | **94.75** |
| **67** | **BM** | **Female** | **50** | **New diagnosed** | **M5** | **6.90** | **52.02** | **NPM1** | **CD56+HLA-DR+CD117+CD33+CD15-CD19-CD38-CD7-** | **83.42** | **98.45** |
| **68** | **BM** | **Female** | **67** | **New diagnosed** | **M2** | **16.13** | **78.81** | **AML1-ETO** | **CD117+CD34+CD33+HLA-DR+CD7+CD13+CD38+CD200+cCD79a+MPO+CD56-CD16-CD11b-CD14-CD64-CD11c-CD15-CD2-CD3-CD10-CD20-CD36-** | **57.47** | **93.02** |
| **69** | **BM** | **Male** | **73** | **New diagnosed** | **M5** | **13.46** | **32.77** | **-** | **CD34+CD117+HLA-DR+CD33+CD13+CD38+MPO+CD64+CD7-CD19-CD56-CD16-CD11b-CD14-CD11c-CD15-CD96-CD36-CD10-cCD3-cCD79a** | **48.99** | **97.94** |
| **70** | **BM** | **Male** | **45** | **New diagnosed** | **M5** | **0.80** | **7.44** | **MLL-AF6** | **CD34+CD33+CD38+CD117+HLA-DR+CD7-CD19-CD56-CD15-CD14-CD11b-CD96-** | **98.89** | **89.52** |
| **71** | **BM** | **Female** | **68** | **New diagnosed** | **M2** | **5.33** | **41.46** | **-** | **CD117+CD34+CD33+HLA-DR+CD19+CD13+CD56-CD7-CD15-CD14-CD96-CD11b-CD16-** | **42.09** | **99.51** |
| **72** | **BM** | **Female** | **45** | **New diagnosed** | **M4** | **3.42** | **16.74** | **-** | **CD117+CD34+CD33+HLA-DR+CD38+CD7+CD19-CD56-CD15-CD14-CD96-CD11b-** | **96.04** | **71.15** |
| **73** | **BM** | **Male** | **76** | **New diagnosed** | **M5** | **3.53** | **78.65** | **DNMT3A; WT1** | **CD117+CD33dim+HLA-DRdim+CD13+CD38+MPO+CD34+CD19-CD56-CD15-CD16-CD11b-CD64-CD11c-CD36-CD4-cCD79a-cCD3-** | **97.68** | **17.05** |
| **74** | **BM** | **Female** | **34** | **Relapse** | **M5** | **0.71** | **47.59** | **NPM1; GATA2; ARID1A; WT1** | **CD34+HLA-DRdim+CD117dim+CD33+CD7-CD19-CD56-CD14-CD96-CD15-CD11b-** | **94.51** | **72.97** |
| **75** | **BM** | **Female** | **48** | **New diagnosed** | **M5** | **166.45** | **52.84** | **ASXL1; FLT3; RUNX1; WT1** | **CD117+CD34+HLA-DR+CD33+CD96+CD13+CD38+CD4dim+CD7+CD19-CD56-CD15-CD14-CD1b-CD16-CD300e-MPO-cCD79a-cCD3-CD10-CD36-** | **95.53** | **94.71** |
| **76** | **BM** | **Female** | **70** | **New diagnosed** | **M4** | **10.72** | **6.06** | **ASXL1; TP53; SF3B1; JAK2** | **CD34+CD117+HLA-DR+CD33+CD13+CD11c+CD38+CD7-CD19-CD56-CD16-CD11b-CD14-CD64-CD15-CD36-CD22-CD10-** | **26.99** | **92.97** |
| **77** | **BM** | **Female** | **32** | **Relapse** | **M2** | **3.42** | **49.99** | **AML1-ETO** | **CD117+HLA-DR+CD33+CD56+CD96+CD19+CD38+CD7-CD15-CD14-CD11b-** | **99.39** | **97.26** |
| **78** | **BM** | **Male** | **56** | **New diagnosed** | **M4** | **1.85** | **35.00** | **DDX41; KRAS** | **CD34+CD117+CD33+HLA-DR+CD13+CD38+MPOdim+CD7-CD19-CD56-CD11b-CD14-CD64-CD300e-CD15-cCD3-** | **87.59** | **49.42** |
| **79** | **BM** | **Male** | **64** | **New diagnosed** | **M5** | **5.01** | **9.80** | **-** | **CD117+CD33+HLA-DRdim+CD13+CD7-CD19-CD56-CD15-CD96-CD16-CD64-CD19-** | **34.36** | **89.32** |
| **80** | **BM** | **Female** | **49** | **Relapse** | **M2** | **29.83** | **83.08** | **ASXL1; NPM1; WT1** | **CD117+CD34+HLA-DRdim+CD33+CD19-CD7-CD38-CD14-CD16-CD64-CD96-MPO-** | **60.82** | **12.32** |
| **81** | **BM** | **Female** | **20** | **New diagnosed** | **M5** | **28.93** | **81.35** | **-** | **CD33bri+HLA-DR+CD56+CD13+CD11b+CD64+CD14+CD11c+CD38+CD15dim+CD45dim+CD7-CD19-CD34-CD117-CD16-** | **2.23** | **83.15** |
| **82** | **BM** | **Female** | **51** | **New diagnosed** | **M4** | **4.00** | **75.83** | **FLT3; NRAS** | **CD117+CD34+HLA-DR+CD33+CD13+CD38+MPO+CD7-CD19-CD56-CD16-CD11b-CD14-CD64-CD11c-CD15-cCD79a-cCD3-** | **24.17** | **75.83** |
| **83** | **BM** | **Male** | **47** | **New diagnosed** | **M5** | **144.13** | **81.95** | **FLT3; NPM1; DNMT3A** | **CD117+HLA-DR+CD33+MPO+CD13+CD38+CD64+CD15+CD7-CD19-CD56-CD16-CD11b-CD14-CD11c-CD36-CD10-cCD79a-cCD3-** | **81.95** | **64.98** |
| **84** | **BM** | **Male** | **58** | **New diagnosed** | **M5** | **16.88** | **56.26** | **-** | **CD117+CD33+HLA-DR+CD13+cMPO+CD34+CD28+CD7-CD19-CD56-CD16-CD11b-CD14-CD64-CD300e-CD15-cCD79a-cCD3-** | **54.24** | **56.26** |
| **85** | **BM** | **Female** | **15** | **New diagnosed** | **M2** | **0.97** | **40.30** | **JAK2** | **CD34+CD117+HLA-DR+CD33+CD13+CD38+MPOdim+CD7-CD19-CD16-CD56-CD11b-CD14-CD64-CD11c-CD15-CD10-CD36-cCD79a-cCD3-** | **89.66** | **33.20** |
| **86** | **BM** | **Male** | **76** | **New diagnosed** | **M5** | **13.32** | **39.68** | **DNMT3A; TP53; WT1;** | **CD117+CD33bri+CD56+HLA-DRdim+CD13+CD7-CD19-CD34-CD16-CD64-CD15-MPO-cCD79a-cCD3-CD38-CD56-CD96-CD36-CD61-CD9-** | **10.81** | **86.73** |
| **87** | **BM** | **Female** | **43** | **Relapse** | **M2** | **2.87** | **86.30** | **CEBPA; TET2** | **CD117+CD34+HLA-DR+CD33+CD96+CD56+CD7-CD19-CD15-CD14-CD11b-** | **74.11** | **98.20** |
| **88** | **BM** | **Male** | **29** | **Relapse** | **M5** | **3.59** | **22.80** | **TET2** | **CD117+CD33+HLA-DRdim+CD34+CD7-CD19-CD56-CD15-CD14-CD11b-CD38-** | **34.89** | **22.80** |
| **89** | **BM** | **Female** | **66** | **Relapse** | **M5** | **29.58** | **88.86** | **-** | **CD117+CD34+CD33+CD7-CD19-CD56-HLA-DR-CD14-CD15-CD11b-** | **95.78** | **83.67** |
| **90** | **BM** | **Male** | **35** | **Relapse** | **M2** | **1.94** | **5.79** | **-** | **CD34bri+CD117+HLA-DR+CD33+CD19+CD56+CD38dim+CD7-CD15-CD11b-CD14-** | **94.92** | **79.06** |

**Supplementary table 2:** We initially determined the expression levels of CD123 and CLL1 in three primary cell lines isolated from peripheral blood samples of these AML patients (n=3). The clinical characteristics of these individuals are depicted.

| **No.** | **Sex** | **Age** | **Subtype** | **Status** | **Blast(%)** | **Cytogenetics Abnormality** |
| --- | --- | --- | --- | --- | --- | --- |
| **1** | Female | 47 | AML-M5 | Relapse | 92.61 | DNMT3A:IDH2 |
| **2** | male | 76 | AML-M5 | Relapse | 86.42 | NPM1;DNMT3A;TET2 |
| **3** | Female | 32 | AML-M2 | Relapse | 49.99 | AML-ETO |
